# Neoplastic and Nonneoplastic Cutaneous Tumors of Dogs in Grenada, West Indies

**DOI:** 10.5402/2011/416435

**Published:** 2011-12-25

**Authors:** A. Chikweto, P. McNeil, M. I. Bhaiyat, D. Stone, R. N. Sharma

**Affiliations:** Pathobiology Academic Program, School of Veterinary Medicine, St. George's University, P. O. Box 7, Grenada, West Indies

## Abstract

This retrospective survey was undertaken between 2002 and 2007 on samples from dogs residing in Grenada. The objectives of the study were to identify the most common histologic types of canine cutaneous tumors, determine the relative frequency of each tumor type, and compare results to reports from other regions. In a series of 225 skin masses examined, the proportion of neoplasms was 72% whereas nonneoplastic tumors accounted for 15.6%, and inflammatory conditions constituted 12.4%. There were 10 types of nonneoplastic tumors with hamartomas being the most common (28.5%), followed by sebaceous hyperplasia (25.7%) and fibroepithelial polyps (22.8%). The 10 most common cutaneous neoplasms were hemangiosarcomas (19.1%), histiocytomas (8.6%), melanocytomas (8%), mast cell tumors (6.8%), lipomas (6.8%), hemangiopericytomas (6.2%), papillomas (5.6%), fibrosarcomas (5.6%), hemangiomas (4.9%), and squamous cell carcinomas (4.3%). Tumors of vascular origin and transmissible venereal tumors were more common in dogs in our study than reported from other regions.

## 1. Introduction

Surveys on skin diseases of dogs demonstrate that neoplastic tumors of the skin are common [1–3]. Several studies report that in dogs the skin is the most commonly affected organ for both neoplastic and nonneoplastic tumors [4–7]. In one study, skin neoplastic and nonneoplastic tumors ranked second only to mammary gland tumors [8].

It has long been recognized among human populations that incidence rates and relative frequencies for several types of neoplasms differ markedly by geographic region [9]. Although studies on cutaneous neoplasms of dogs from different geographic regions report similarities, differences in tumor types and relative frequencies have also been reported. Such studies include those from the United Kingdom [10]; Greece [11]; United States of America [6]; Australia [12]; Korea [13]; Thailand [7]; Brazil [14]; Zimbabwe [15]; India [16]; Zambia [17]. For example, cutaneous histiocytoma was the most frequently reported neoplasm in dogs in India and Zambia [16, 17]; lipoma was the most common skin tumor in dogs in Korea [13], and mast cell tumor ranked first in studies on dogs from the United States, Thailand, the United Kingdom, Greece, Brazil, and Zimbabwe [6, 7, 10, 11, 14, 15]. Transmissible venereal tumor in the skin was only reported in studies from Korea and Brazil [13, 14]. Studies on canine nonneoplastic skin tumors have also reported differences in types and in frequency of these tumors. For example, studies from Korea, Thailand, and Brazil report that epidermal and follicular cysts are the most common nonneoplastic tumors affecting the skin of dogs followed by sebaceous hyperplasia, whereas a study from Serbia reports only the occurrence of dermoid cysts [7, 8, 13, 14].

Although geographic differences for the types and frequencies of canine cutaneous neoplastic and nonneoplastic tumors are recognized, the reasons for these differences are not well understood. For some types of cutaneous tumors it is likely that both climate and the breeds of dogs in respective locations play a role. Ultraviolet radiation from sunlight has been implicated as a risk factor for canine cutaneous hemangiosarcoma, hemangioma, and squamous cell carcinoma [18, 19]. The Boxer breed of dog is more susceptible to mast cell tumors [20]. Importantly, none of the studies on the frequencies of canine skin tumors to date reflect data from tropical island communities such as exist in the Caribbean. We hypothesize that in Grenada, a small island state in the Caribbean with a warm, humid climate (mean temperature of 28°C/82°F) and a relatively confined, interbreeding mongrel dog population that the types and relative frequencies of canine skin tumors are different from that reported elsewhere. To our knowledge, this study is the first survey of canine skin tumors in a small tropical island setting.

The objectives of this retrospective study were to identify the most common histologic types of cutaneous neoplastic and nonneoplastic canine tumors, determine the relative frequency of each tumor type, and compare the results with published findings from different geographic regions.

## 2. Materials and Methods

The island of Grenada is bordered by the Caribbean Sea to the West and the Atlantic Ocean to the East. Its total area is about 340 km^2^. The island has equable temperatures varying slightly with altitude and averaging 82°F (28°C).

Canine skin biopsies and samples from necropsy cases with skin masses suspected to be neoplastic processes and submitted to the diagnostic pathology laboratory from 2002 to 2007 were included in this study. Samples were submitted from the Small Animal Hospital, the Grenada Society for Prevention of Cruelty to Animals and from private clinics. Samples were submitted fixed in 10% buffered formalin, processed, sectioned at 3 *μ*m, stained with hematoxylin and eosin, and examined microscopically.

 Tumors were diagnosed and classified according to the current World Health Organization (WHO) classification of animal tumors [21–23]. Where possible, history relating to sex, breed, age, and site of the tumor on the body was obtained along with the submission. Location of the tumor on the body was categorized into 4 groups: head and neck, trunk, limbs, and multiple sites.

In this study, masses were grouped according to neoplastic, nonneoplastic, and inflammatory. We defined nonneoplastic tumors as neoplastic-like lesions without an inflammatory component whereas masses with inflammatory infiltrates with/or without an intralesional etiologic agent were defined as inflammatory lesions.

In addition, published studies on skin tumors of dogs from other geographic regions were evaluated and compared with the results of our study.

## 3. Results

Two hundred and twenty five canine skin masses from 207 dogs comprising 89 females and 118 males were examined. Among these skin masses, the prevalence of neoplasms was 72% (95% confidence interval, 66.13% to 77.87%) (162/225). Thirty-five (15.6%) (95% CI, 10.82% to 20.3%) were diagnosed as nonneoplastic tumors, and 28 (12.4%) (95% CI, 8.13% to 16.75%) were inflammatory conditions. Inflammatory conditions included pyogranulomatous dermatitis (50%); nodular and diffuse chronic dermatitis (25%); deep and superficial pyoderma (10.7%); sterile granuloma (10.7%), and allergic dermatitis (3.6%). Seventeen dogs had more than one type of tumor, excluding inflammatory lesions.

More than half of the samples (58%) were from the mixed local breed of dog referred to as the Grenadian pothound. Pothounds are the most common type of dog in Grenada. They are often owned but generally allowed to wander at will. Other breeds represented in this study in descending order of frequency were German Shepherd Dog, Rottweiler, Boston Terrier, Jack Russell Terrier, Scottish Terrier, Pompek, Rhodesian Ridgeback, Labrador Retriever, Boxer, Golden Retriever, Shih Tzu, Dachshund, Doberman Pinscher, Bulldog, Pit bull Terrier, Bouvier, and Anatolian Shepherd Dog.

 The frequency, mean age in years, and male-to-female ratio of each neoplastic and nonneoplastic tumor type are as shown in Table 1. Among the 162 neoplastic skin masses examined, 27 histologic types were diagnosed. The 10 most common neoplasms were hemangiosarcomas 31 (19.1%), histiocytomas 14 (8.6%), melanocytomas 13 (8%), mast cell tumors 11 (6.8%), lipomas 11 (6.8%), hemangiopericytomas 10 (6.2%), papillomas 9 (5.6%), fibrosarcomas 9 (5.6%), hemangiomas 8 (4.9%), and squamous cell carcinomas 7 (4.3%). Among the 10 most common neoplasms, the type of neoplasm and prevalence in each of the two breed categories (local mixed breeds and other breeds) is as shown in Table 2.

There were 10 types of nonneoplastic tumors including sebaceous hyperplasia 9 (25.7%), fibroepithelial polyps 8 (22.9%), collagenous hamartomas 4 (11.45%), infundibular cysts 4 (11.45%), fibroadnexal hamartomas 3 (8.6%), scrotal vascular hamartomas 2 (5.7%), isthmus cysts 2 (5.7%), sebaceous hamartomas 1 (2.8%), dermoid cysts 1 (2.8%), and fibropruritic nodules 1 (2.8%).

 Among the neoplastic tumors, 62 (38.3%) were malignant whereas 100 (61.7%) were benign. Generally, neoplasms occurred in relatively older dogs with a mean age of 7.6 years. Neoplastic tumors were most frequently located on the trunk followed in descending order by limbs, head and neck, and multiple sites. Among nonneoplastic tumors limbs were the most frequently affected site followed by trunk, head and neck, and multiple sites.

Of the 4 cases of cutaneous transmissible venereal tumor (TVT) in this study, 3 were diagnosed in castrated males and one was diagnosed in an intact female. It was not clear from the records if the dogs were castrated prior to presentation or at the time of tumor removal. Three of the 4 TVT cases had histologic evidence of metastasis. Hepatoid (perianal) gland tumors were diagnosed in 2 male dogs of unknown neuter status and in 1 spayed female.

## 4. Discussion

Results from our study confirm that the types of skin tumors affecting dogs in Grenada are consistent with previous reports from other geographic areas but the relative frequency of skin tumor types differ. In our study neoplastic tumors represented 72% (95% CI 66.13% to 77.87%) of all skin masses (neoplastic, nonneoplastic, and inflammatory). This is within the range of other published studies conducted in Zimbabwe, Brazil, and India where neoplastic tumors represented 60%, 88%, and 95%, respectively of all skin tumors [14–16]. However, when the frequency of neoplastic tumor types is compared, important differences are noted (Table 3). In our study hemangiosarcoma was the most commonly reported canine skin neoplasm. In other studies the most commonly reported skin neoplasm was the mast cell tumor except for Korea where lipoma was more frequently reported [13] and India and Zambia where histiocytoma was the most commonly reported skin neoplasm [16, 17]. Our study is the first to report hemangiosarcoma as the most commonly diagnosed canine skin neoplasm, comprising 19.1% of all cutaneous neoplasms evaluated. Adhering to the 1998 WHO classification of tumors of vascular tissue [21], a diagnosis of hemangioma was restricted to well-circumscribed tumors consisting of vascular channels lined by well-differentiated cells. Locally infiltrative lesions with irregular vascular channels were classified as hemangiosarcomas. This differs from the approach taken by Hargis et al. where greater emphasis was placed on the presence of mitotic activity such that even locally infiltrative lesions were classified as hemangiomas [18]. In dogs it is now generally accepted that solar radiation is a contributing factor in the development of several skin neoplasms including hemangiomas and hemangiosarcomas [18–21]. Indeed, Gross et al. recognize a subset of vascular tumors as solar-induced dermal hemangiomas [20]; they note the difficulties in distinguishing benign tumors from malignant and suggest that there is a continuum from vascular ectasia to benign and malignant neoplasia. In our study the majority of the vascular growths were located on sparsely haired areas such as the ventral abdomen, inguinal areas, and limbs. This lends support to a role of sun exposure in the pathogenesis of these skin neoplasms, as sparsely haired skin is more exposed to the harmful effects of solar radiation than haired areas. Additionally, in the adjacent nonneoplastic areas of some of these neoplasms we noted other changes that may be associated with solar radiation, including vascular ectasia (18 dogs), mild superficial dermal fibrosis (11 dogs), collagen degeneration or solar elastosis (4 dogs), pigmentary incontinence (3 dogs), and hyperkeratosis (2 dogs). Importantly, there is abundant sunshine in Grenada year around, and most dogs in Grenada live outdoors their entire lives. This is in direct contrast with the situation in the Greek study where 2.9% of 174 neoplasms were hemangiosarcomas and 3.4% were hemangiomas; the authors noted that the majority of dogs in their area spent most of their time indoors [11]. Genetics should also be considered as a possible risk factor for these vascular skin tumors in dogs, as inherited forms of cutaneous angiomatosis in humans have been documented [24], and in dogs there is a breed predisposition for scrotal vascular hamartomas [25].

Although not in the top 10 most common skin neoplasms, the frequency of cutaneous TVT was high in our study compared to other studies, comprising 2.5% of all skin neoplasms. The majority of the published studies reviewed did not report any TVT in the skin of dogs. Only studies from Brazil and Korea reported cutaneous TVT and in both of these studies cutaneous TVT represented less than 0.6% of skin neoplasms out of over 600 cutaneous neoplasms evaluated in each study [13, 14]. The higher frequency of cutaneous TVT in dogs in Grenada may reflect uncontrolled breeding as a result of unrestricted movement of unneutered dogs. It is also possible that the pothound, the predominant dog type in Grenada, is at increased risk for cutaneous TVT, as was suggested in a previous report [26].

All but one of the studies evaluated for comparison to our results had mast cell tumors ranked first in relative frequency for skin neoplasms (Table 3). In our study mast cell tumors were ranked 4th. It has been reported that certain breeds of dogs, including Boxers, Labradors, and Terriers, are at increased risk for mast cell tumors [10, 20]. The lower ranking for frequency of mast cell tumors in our study is likely due to few pure breed dogs known to be at increased risk for this tumor type in our study population.

 Most histiocytomas (60–80%) occur in dogs less than 5 years of age, though they can be found throughout life [27]. In our study age was known for 12 of the 14 dogs diagnosed with histiocytoma. Dogs ranged in age from 3 months to 14 years. Of these 12 dogs 7 were under 5 years of age with 4 of these being under 2 years of age. Importantly, 3 dogs were 10 years or older. A few older dogs in our small sample size for this tumor type resulted in a mean age of 4.7 yrs. Several other studies also report a mean age similar to our observation. In a study from Australia the mean age was 3.9 years (range 5 months to 13 years); in a study from Zimbabwe the mean age was 5.45 years (range 0.6 to 12 years); in a study from Thailand the mean age was 5.13 years (range 3 months to 15 years) [7, 12, 15]. It is possible that the age distribution in our study reflects a delay in diagnosis due to the fact that routine and preventive veterinary care for dogs is not the norm in Grenada.

To our knowledge this is the first study detailing the types and frequencies of neoplastic and nonneoplastic skin tumors in dogs from Grenada. One limitation of this study is that it does not include samples from dogs which were not evaluated by a veterinarian. Nor does it include samples from dogs seen by veterinarians who did not request histologic evaluation of skin masses. These limitations are likely a component of most studies of canine tumors. Thus, comparison of relative frequencies of the tumor types in this study to other studies has merit and helps to shed light on possible risk factors for specific tumors. In our study the Grenadian pothound is the most frequently represented type of dog diagnosed with skin tumors, reflecting that the vast majority of dogs in Grenada are pothounds. It is possible that the differences we observed in the Grenada dog population for relative frequencies on skin neoplasms compared to other published reports is due to pothounds being genetically more susceptible to some tumors and more resistant to others. It is also possible that environmental factors may account for and/or contribute to the differences we observed. Grenada represents a geographic area with a set of environmental conditions distinct from locations of other published reports of skin tumors in dogs. Possible environmental risk factors for skin tumors in Grenada include abundant solar radiation, year around warm, humid climate, and heavy ectoparasite loads and associated dermatitis in many dogs.

## 5. Conclusions

Our results provide valuable information on specific tumor types observed at increased frequency within this unique tropical climate. This information on cutaneous tumors in dogs may serve as a reference for future comparative studies in Grenada and the Caribbean region. Because dogs are the one domestic animal that most closely shares the human environment, determining environmental risk factors for canine tumors may contribute to identification and understanding of such risk factors for similar tumors in humans.

## Figures and Tables

**Table 1:** Histological types, frequency, mean age (in years), and sex ratio for neoplastic and nonneoplastic tumors in the skin of dogs from Grenada, West Indies.

Tumor type	Frequency	Mean age (yrs)	M : F
*Hematopoietic tumors (n* = 4)			
Indolent plasmacytoma	3	7.3	2 : 1
Lymphoma	1	6.0	0 : 1
*Epithelial and melanocytic tumors (n* = 80)			
(i) Tumors of the epidermis			
Papilloma	9	6.1	5 : 4
Squamous cell carcinoma	7	6.3	5 : 2
(ii) Tumors with adnexal differentiation			
Infundibular keratinizing acanthoma	1	10.0	1 : 0
Trichoepithelioma	3	7.6	1 : 2
Trichoblastoma	4	6.0	3 : 1
(iii) Sebaceous and modified sebaceous gland tumors			
Hepatoid gland adenoma	2	10.5	1 : 1
Hepatoid gland carcinoma	1	12.0	1 : 0
Sebaceous epithelioma	1	13.0	1 : 0
Sebaceous adenoma	1	10.0	0 : 1
(iv) Apocrine and modified apocrine tumors			
Apocrine adenoma	1	13.0	1 : 0
Apocrine adenocarcinoma	2	N. R	1 : 1
(v) Nail bed tumors			
Subungual keratoacanthoma	1	2.5	1 : 0
(vi) Melanocytic tumors			
Melanocytoma	13	6.9	9 : 4
Malignant melanoma	5	8.9	3 : 2
(vii) Cysts			
Infundibular cyst	4	5.6	2 : 2
Dermoid cyst	1	N. R*	1 : 0
Isthmus cyst	2	8.5	1 : 1
(viii) Hamartomas			
Sebaceous hamartoma	1	13	1 : 0
Fibroadnexal hamartoma	3	10	2 : 1
(ix) Tumor-like lesions			
Sebaceous hyperplasia	9	9.8	5 : 4
Fibroepithelial polyp	8	7.4	2 : 6
Fibropruritic nodule	1	4	1 : 0
*Mesenchymal tumors (n* = 99)			
(i) Tumors of vascular tissue			
Hemangioma	8	8.9	6 : 2
Scrotal vascular hamartoma	2	6.0	2 : 0
Hemangiosarcoma	31	8.3	19 : 12
(ii) Histiocytic tumors			
Histiocytoma	14	4.7	7 : 7
Malignant histiocytosis	4	8.0	2 : 2
(iii) Mast cell tumors	11	7.3	8 : 3
(iv) Tumors of fibrous tissue			
Fibroma	3	7.2	1 : 2
Collagenous hamartoma	4	7.6	2 : 2
Fibrosarcoma	9	6.7	3 : 6
(v) Tumors of adipose tissue			
Lipoma	11	8.7	4 : 7
Liposarcoma	2	8.5	1 : 1
*Unclassified tumors (n* = 14)			
Hemangiopericytoma	10	7.7	5 : 5
Transmissible venereal tumor	4	5.5	3 : 1

*N. R: Age not reported; M: Male; F: Female.

**Table 2:** Breed distribution with respect to the top 10 neoplasms in the skin of dogs from Grenada, WI.

Type of neoplasm	Local mixed breed	Other breeds*
Hemangiosarcoma	22 (71.0%)	9 (29.0%) BX (2), LB (4), DB (1), RT (1), JR (1)
Histiocytoma	12 (85.7%)	2 (14.3%) GSD (1), ST (1)
Melanocytoma	10 (77.0%)	3 (23.0%) LB (2), DB (1)
Mast cell tumor	5 (45.5%)	6 (54.5%) BT (2), RT (1), JR (1), BD (1), GR (1)
Lipoma	6 (54.5%)	5 (45.5%) DB (2), BV (1), RB (1), RT (1)
Hemangiopericytoma	6 (60.0%)	4 (40.0%), RB (3), GR (1)
Papilloma	6 (66.7%)	3 (33.3%) PK (3)
Fibrosarcoma	6 (66.7%)	3 (33.3%) RT (1), DB (1), GSD (1)
Hemangioma	4 (50.0%)	4 (50.0%), DH (1), JR (1), PK (1), BX (1)
Squamous cell carcinoma	2 (28.6%)	5 (71.4%), PB (1), LB (1), DB (1), GSD (1), GR (1)

Total	79 (64.2%)	44 (35.8%)

*GR: Golden Retriever; BD: Bulldog; ST: Scottish Terrier; RB: Rhodesian Ridgeback; BV: Bouvier; PK: Pompek; DH: Dachshund, JR: Jack Russell Terrier; PB: Pitbull; DB: Doberman Pinscher; LB: Labrador Retriever; GSD: Germany shepherd Dog; BT: Boston Terrier; RT: Rottweiler; BX: Boxer.

**Table 3:** Relative frequency (%) of the 10 most common neoplasms in a series of 162 cutaneous neoplasms from dogs in Grenada compared to the published results of surveys from other countries.

Tumor	Grenada	Zimbabwe [15]	Thailand [7]	Korea [13]	U K [10]	Greece [11]	Australia [12]	USA [6]	Brazil [14]
	*n* = 162	*n* = 540	*n* = 1231	*n* = 605	*n* = 2616	*n* = 174	*n* = 1000	*n* = 948	*n* = 673
Hemangiosarcoma	19.1	4.6	0.8	N.R*	N.R*	2.9	4.3	1.5	3.7
Histiocytoma	8.6	2.4	3.7	9.3	6.0	5.7	12.6	2.5	2.9
Melanocytoma	8.0	5.0	7.6	2.9	6.3	1.1	1.8	5.0	1.5
Mast cell tumor	6.8	20.3	23.8	10.9	19.2	13.8	16.1	21.3	23.3
Lipoma	6.8	4.1	3.4	14.1	8.5	5.7	6.0	8.6	6.1
Hemangiopericytoma	6.2	3.2	2.5	2.0	4.2	2.3	7.3	3.2	2.1
Papilloma	5.6	2.2	3.2	2.6	2.3	3.4	0.2	2.5	4.4
Fibrosarcoma	5.6	4.2	3.0	N.R	7.4	4.2	6.6	5.9	1.5
Hemangioma	4.9	2.2	2.9	1.3	2.4	3.4	3.7	3.0	3.7
Squamous cell carcinoma	4.3	15.4	6.7	0.2	5.4	2.3	6.9	3.9	7.8

*N. R: Not reported.
